# Supplementary material for: Pain after laparoscopic endometriosis-specific vs. hysterectomy surgeries: A retrospective cohort analysis
Source: PLoS One. 2024 Oct 4;19(10):e0301074. doi: 10.1371/journal.pone.0301074 (PMC11452001; doi:10.1371/journal.pone.0301074)
Supplement: Attachment — Submitted filename: Revision .docx [file pone.0301074.s001.docx]

The manuscript proposed by the Author appears to be original and interesting, but to be accepted for publication, some sections of the study must be improved and implemented:

-In the introduction I suggest focusing better on the concept of pain sensitization by also citing works such as this "Prevalence and Risk Factors of Central Sensitization in Women with Endometriosis", Raimondo D et al. 2023

-in the materials and methods section there is no description of the surgical technique and above all the extent of surgery for endometriosis (were visceral resections performed? Parametrectomies?). These aspects must therefore also be specified in a descriptive table in the "results" section

-The discussion section should be significantly expanded, also discussing the role that the surgical procedures performed in the group of patients operated for endometriosis may have had in the use of any opioids in the post-operative period. It is important in this section to discuss the possible role of parametrectomy in endometriosis surgery ("Impact of nerve-sparing posterolateral parametrial excision for deep infiltrating endometriosis on postoperative bowel, urinary, and sexual function", Ianieri et al 2022) and of modified radical hysterectomy with parametrectomies for endometriosis (“Surgical and functional impact of nerve-sparing radical hysterectomy for parametrial deep endometriosis: a single center experience”, Rosati A et al, 2022).

Furthermore, it could be interesting to understand whether patients with functional disorders linked to pelvic floor disfunction may need greater use of pain-relieving therapy in the post-operative period ("Pelvic floor dysfunction at transperineal ultrasound and chronic constipation in women with endometriosis", Raimondo D et al 2022)
